# Association between Recruitment Methods and Attrition in Internet-Based Studies

**DOI:** 10.1371/journal.pone.0114925

**Published:** 2014-12-09

**Authors:** Paolo Bajardi, Daniela Paolotti, Alessandro Vespignani, Ken Eames, Sebastian Funk, W. John Edmunds, Clement Turbelin, Marion Debin, Vittoria Colizza, Ronald Smallenburg, Carl Koppeschaar, Ana O. Franco, Vitor Faustino, AnnaSara Carnahan, Moa Rehn, Franco Merletti, Jeroen Douwes, Ridvan Firestone, Lorenzo Richiardi

**Affiliations:** 1 GECO- Computational Epidemiology Group, Department of Veterinary Sciences, University of Torino, Torino, Italy; 2 ISI Foundation, Turin, Italy; 3 Laboratory for the Modeling of Biological and Socio-technical Systems Northeastern University, Boston, Massachusetts, United States of America; 4 Institute for Quantitative Social Sciences at Harvard University, Cambridge, Massachusetts, United States of America; 5 London School of Hygiene and Tropical Medicine, London, United Kingdom; 6 Institut National de la Santé et de la Recherche Médicale, UMR-S 707, Paris, France; 7 Université Pierre et Marie Curie-Paris 6, UMR-S 707, Paris, France; 8 Aquisto-Inter BV, Amsterdam, The Netherlands; 9 Instituto Gulbenkian de Cincia, Oeiras, Portugal; 10 Public Health Agency of Sweden, Stockholm, Sweden; 11 Department of Medical Sciences, University of Turin and CPO-Piemonte, Turin, Italy; 12 Centre for Public Health Research, Massey University, Wellington, New Zealand; Örebro University, Sweden

## Abstract

Internet-based systems for epidemiological studies have advantages over traditional approaches as they can potentially recruit and monitor a wider range of individuals in a relatively inexpensive fashion. We studied the association between communication strategies used for recruitment (offline, online, face-to-face) and follow-up participation in nine Internet-based cohorts: the Influenzanet network of platforms for influenza surveillance which includes seven cohorts in seven different European countries, the Italian birth cohort Ninfea and the New Zealand birth cohort ELF. Follow-up participation varied from 43% to 89% depending on the cohort. Although there were heterogeneities among studies, participants who became aware of the study through an online communication campaign compared with those through traditional offline media seemed to have a lower follow-up participation in 8 out of 9 cohorts. There were no clear differences in participation between participants enrolled face-to-face and those enrolled through other offline strategies. An Internet-based campaign for Internet-based epidemiological studies seems to be less effective than an offline one in enrolling volunteers who keep participating in follow-up questionnaires. This suggests that even for Internet-based epidemiological studies an offline enrollment campaign would be helpful in order to achieve a higher participation proportion and limit the cohort attrition.

## Introduction

The pervasiveness of the Internet represents an unprecedented opportunity to devise potentially massive and relatively inexpensive epidemiological studies. Voluntary participation, as well as recruitment through a dedicated web site, is easy to manage and advertise. In the last few years we have witnessed increasing interest in Internet-based study design, including in methodological research on how to use the Internet to recruit and follow-up participants in cohort studies [1]–[9].

Enrollment of a convenience sample using the Internet restricts the source population (to Internet users) and generates a self-selected study population. In this paper we will not discuss issues of validity related to this baseline selection [1], [10], [11] but we will study follow-up participation and, in particular, whether the different strategies to communicate and advertise the existence of the study and to enroll participants are related to compliance with subsequent follow-up.

We will focus on nine different Internet-based cohort studies: Influenzanet (www.influenzanet.eu), a European network including seven national influenza-like-illness surveillance platforms; the Ninfea study (www.progettoninfea.it), a multi-purpose Italian Internet-based birth cohort; and the ELF study (www.elfs.org.nz), a similar multi-purpose birth cohort set up in New Zealand. These studies differ in the target populations (general population vs. pregnant women), the investigated outcomes (infectious vs. chronic diseases), the length of follow-up (weeks-months vs. months-years), as well as the country in which they have been conducted. This heterogeneity is helpful to improve the generalizability of our findings to Internet-based studies and to identify potential study-specific effects.

## Materials and Methods

### Ethics Statement

The Flusurvey study (UK) was approved by the London School of Hygiene & Tropical Medicine Ethics Committee (Application number 5530). The Influensakoll study (SE) was approved by the Stockholm Regional Ethical Review Board (Dnr. 2011/387-31/4). The Grippenet study (FR) was approved by the CCTIRS advisory committee on information processing for research, authorization 11.565 and by the CNIL, French Data Protection Authority, authorization DR-2012-024. In all the participating countries, the study was conducted in agreement with national regulations on privacy, data collection and treatment. Informed consent has been obtained from individuals who participated in the study.

The Ninfea study was approved by the Ethical Committee of the SanGiovanni Battista Hospital-CTO/CRF/Maria Adelaide Hospital of Turin (protocol 0048362, 2005).

Ethical approval for the ELF study was obtained in 2007 from Massey University Human Ethics Committee, New Zealand (MUHEC Application 07/62).

### Cohort studies

#### Influenzanet cohorts

Influenzanet is a network of country-wide Internet-based systems used to monitor influenza-like-illness (ILI) in several European countries with the aid of self-selected volunteers [12]–[16]. It has been operational in the Netherlands and Belgium since 2003, in Portugal since 2005, in Italy since 2008, in the United Kingdom since 2009 and in France and Sweden since 2011.

The present study is based on the activity of volunteers registering and reporting their symptoms during the 2011–2012 influenza season. During this period, researchers in charge of each national platform were responsible for promoting the study in their own country in order to enroll as many individuals as possible. Because of this, different strategies were applied in the different countries, depending on the availability of resources and promotion opportunities. In general, it was attempted to generate publicity through other websites, ranging from health-related portals to online general newspapers. Occasional TV and radio interviews by members of the different Influenzanet national teams brought great attention to the national websites. Some local initiatives involved activities in schools or science shows and announcements during public science-oriented conferences.

Upon registration, new participants were invited to complete a short baseline questionnaire in which socio-demographic and lifestyle information was recorded. During the influenza season, volunteers were sent a weekly email newsletter containing an invitation to visit the platform to report their symptoms or the absence of symptoms.

The sample in this study consists of all the Influenzanet volunteers in the seven European countries who contributed with at least one symptoms questionnaire during the 2011–2012 influenza season. Since participants can also create accounts on behalf of other people of their family/household, thus enabling, for instance, adults to report symptoms data for their children or their parents, we discarded participants whose information was reported to have been provided by someone else (e.g. a child whose questionnaires were filled in by a parent). Likewise, we did not consider subjects aged less than 15 or more than 70 years old, on the assumption that a different participant may have managed their account.

We considered that participants were enrolled in the Influenzanet cohort, and therefore eligible for this analysis, if they completed their first symptoms survey at least 60 days before the end of the monitoring season, and filled in at least one more symptoms survey within 15 days of the first symptoms survey. From those enrolled, we considered as ‘participants at follow–up’ those individuals who filled in at least two symptoms surveys during the time window of 30 to 60 days after the first symptoms survey. Otherwise, the enrolled volunteers were considered lost at follow-up.

#### Ninfea cohort

The Ninfea birth cohort study is an ongoing study started in 2005. Detailed information on the study design is available elsewhere [17]. Briefly, the Ninfea cohort enrolls pregnant women who are Internet users and who at any time during their pregnancy decide to register on project website and complete the first baseline questionnaire (Q1). This questionnaire includes questions about socio-economic status, lifestyle, possible environmental and occupational exposures, medical and reproductive history and questions about health status in the first three months of pregnancy. The existence of the Ninfea study was disseminated in collaboration with health personnel who distributed leaflets and/or introduced the study to pregnant women when they visited hospitals or family clinics. Addintionally, in similar settings, posters with the study description were exhibited. Moreover, the study has occasionally attracted local and national media coverage. Finally, hyperlinks to the study website were posted on the home page of selected hospitals, on websites dedicated to pregnant women and on websites of other health-related Internet-based projects (influweb.it, mammainforma.it); on discussion forums related to pregnancy and on the Ninfea page on Facebook [18]. The study requests the completion of two main follow-up questionnaires at 6 (Q2) and 18 (Q3) months after childbirth. After the 18-month questionnaire, women were followed-up using both active (short questionnaires every 2–3 years) and passive (linkage with health related databases) methods.

Women were contacted via email, telephone, text messages or regular mail every time they were supposed to complete a follow-up questionnaire. For the purpose of this study, we restricted the analyses to participation at Q2 in relationship with baseline characteristics of the volunteers obtained during pregnancy at Q1. We restricted the analysis to the first pregnancy for those women who participated in the Ninfea cohort with more than one pregnancy to avoid correlated outcomes. The number of unique women registered in the Ninfea database version 2012.03 (downloaded on March 2012) was 4,617; among these, we selected as eligible for the present study the 3,190 participants whose child was expected to be at least 12 months old by July 2012 (when we set the administrative censure of the study). Among these women we considered as participants at follow-up those who replied to the Q2 online questionnaire within a period of 6-months from the time when they were first contacted to fill in that questionnaire.

#### ELF study

The Early Life Factors (ELF) study is a birth cohort study started in New Zealand in 2008 to explore the relationship between early life exposures and the development of diseases later in life. Up to the date of the current analyses (July 2012) the study includes 1,025 pregnant women. The study design is similar to the above-mentioned Ninfea cohort. The ELF study recruited pregnant women at any time of their pregnancy actively informing them about the study at ‘parent and child shows’ located in the main urban regions. Typically, a parent and child show is a large-scale event, marketed at expecting and experienced parents. People pay to attend these shows because it is a one-stop-shop to purchase standard and newly available products (e.g. food), services (e.g. child-care), recreation and education programs (e.g. developmental courses). The shows are attended by more than 22,000 people annually. The ELF study used other recruitment avenues including: information inserted in antenatal care booklets, promotional posters in hospitals and sonography clinics. Participants could be enrolled through the Internet, accessing directly the study webpage (e.g. after a Google search on pregnancy-related issues) or following the link provided by other participants in online forums. The main difference from the Ninfea cohort is that volunteers were offered the option to participate through traditional postal questionnaire (offline), particularly if they could not participate through the online platform, e.g. because they did not have access to the Internet. Registration consisted therefore of filling an online or posted detailed questionnaire with background information (Q1). Follow-up questionnaires in the ELF study were required to be completed 3-months (Q2), 15 months (Q3) and 2 years (Q4) after childbirth. Participants were reminded via email or regular mail whenever it was time to complete a new questionnaire. Similarly to the Ninfea study, here we focus on the analysis of the first follow-up questionnaire (Q2), and we restrict our analysis to the first pregnancy of those women who participated with more than one pregnancy and we included only women whose children were expected to be at least 12 months old (n = 795) by July 2012. We considered as participants at follow-up those women who completed the questionnaire within 9 months after their initial invitation to respond to that questionnaire.

### Statistical analysis

The impact of different communication strategies was assessed considering three broad classes: online media, offline media, and face-to-face communication. In all the considered studies, at registration volunteers were asked a multiple-choice question about how they had been informed about the study. Based on this information, we created the following mutually exclusive categories of recruitment:

1. *Face-to-face*, included conferences and word of mouth (Influenzanet), information obtained at pre-delivery courses, at family planning clinics, from gynaecologists or word-of-mouth (Ninfea), information from general practitioners, word-of-mouth, parent and child shows (ELF);
2. *Offline media*, included interviews and mentions on television, radio, newspapers and others (Influenzanet), poster, leaflets and others (Ninfea), New Zealand antenatal care booklet (ELF) and excluded every face-to-face communication;
3. *Online methods,* included those who have been aware of the study only through online media, such as links on external websites.

As discussed in the previous section, the ELF study is not exclusively internet-based. Results for the ELF study were obtained considering volunteers that used either the website or the ordinary paper mail to submit the baseline questionnaire, and an additional analysis was performed considering only the individuals who used the website to submit the first questionnaire.

To estimate the association between the communication strategy and participation at follow-up a multivariable logistic regression was performed in each cohort separately. We estimated unadjusted odds ratios (ORs) and ORs adjusted for a number of a-priori selected variables:

age, gender, smoking, educational level, presence of chronic disorder/condition, household composition (adults only or family with children/teenagers [≤18 years old]), vaccination against seasonal influenza (Influenzanet).
age, smoking, educational level, history of chronic diseases/conditions, trimester of pregnancy at the time of registration on the website (Ninfea).
age, smoking, educational level, history of chronic diseases/conditions (ELF).

In the multivariate analysis, we used a complete-case approach, thus excluding individuals who did not provide information about all the variables included in the analysis, which led to the exclusion of 4,681 (17%) subjects from the Influenzanet cohorts, 343 (11%) subjects for the Ninfea cohort and 16 (2%) subjects from the ELF cohort. S1 Table reports the percentage of missing data for each cohort as well as the percentage of participation at follow-up for individuals with complete or missing data.

We carried out a sensitivity analysis restricting the Influenzanet cohort to women aged 18–44 (fertile age), the population used in Ninfea and ELF. Finally, in order to provide an overall synthetic measure of the association between recruitment methods and participation at follow-up, a meta-analytic estimate (random effect) is reported. We also reported the value of i^2^ which indicates the percentage of total variation across cohorts that is due to heterogeneity rather than chance.

## Results

Characteristics of the cohorts and participation proportion at follow-up are summarized in Table 1. Cohorts differ considerably in terms of the sample size (from less than one thousand to more than twelve thousand) as well as the participation at follow-up (ranging between 43% and 89%) and Internet penetration in the different countries (ranging from 55% to 92%).

**Table 1:** Data summary by cohort and country.

Cohort	Country	1Internetpervasiveness	Targetpopulation	Cohortsize*	Study periodfor the presentanalysis	Participants atfollow-up*
Influenzanet	Sweden	91%	Generalpopulation	2,097	Nov. 2011–Mar. 2012	43%
	UK	82%	Generalpopulation	2,171		54%
	Netherlands	92%	Generalpopulation	12,514		77%
	Belgium	78%	Generalpopulation	3,834		79%
	France	80%	Generalpopulation	3,540		63%
	Italy	57%	Generalpopulation	1,354		49%
	Portugal	55%	Generalpopulation	1,152		68%
Ninfea	Italy	57%	Pregnantwomen	3,190	Jul. 2005–Jul. 2012	89%
ELF	NewZealand	86%	Pregnantwomen	795	Sept. 2008–Jul. 2012	62%

*Cohort size = Number of enrolled participants eligible for this study (see text for details regarding the inclusion criteria). Participants at follow up (%) = number of participants at follow-up divided by cohort size (x100).

1 Data referred to the percentage of Internet users in 2011 and were gathered from the International Telecommunication Union (ITU, www.itu.int), the United Nations specialized agency for information and communication technologies.

The number of participants enrolled via each communication category for the various cohorts and corresponding OR estimates are reported in Table 2. Online recruitment appeared to be largely used in the Influenzanet cohort where, on average, 60% of the volunteers had found out about the project web page through another website. In the Ninfea cohort, 16% of individuals became aware of the study from the Internet, and for the ELF study the corresponding figure is 2%.

**Table 2:** Follow-up participation proportion, unadjusted odds ratios and odds ratios adjusted for the variables detailed in the Methods section and confidence intervals are shown for different recruitment methods of individuals enrolled in the Influenzanet study (stratified by country), Ninfea and ELF.

Cohort	Country(complete-casesamplesize*)	Communication strategy(%)	Participants atfollow-up (%)	Lost tofollow-up(%)	OR(unadjusted)	95% CI	OR(adjusted)*	95%CI*
Influenzanet	Sweden(1,807)	offline (28)	221 (44)	284 (56)	1.00		1.00	
		face-to-face (20)	199 (55)	165 (45)	**1.55**	**1.18,2.03**	**1.61**	**1.20,2.14**
		online (52)	360 (38)	578 (62)	**0.80**	**0.64,0.99**	0.88	0.70,1.12
	UK(1,920)	offline (20)	277 (73)	104 (27)	1.00		1.00	
		face-to-face (48)	564 (61)	367 (39)	**0.58**	**0.44,0.75**	0.79	0.59,1.04
		online (32)	215 (36)	393 (64)	**0.21**	**0.15,0.27**	**0.28**	**0.21,0.38**
	Netherlands(10,935)	offline (14)	1,247 (80)	311 (20)	1.00		1.00	
		face-to-face (18)	1,478 (77)	442 (23)	**0.83**	**0.71,0.98**	0.96	0.81,1.13
		online (68)	5,741 (77)	1,716 (23)	**0.83**	**0.73,0.95**	0.90	0.78,1.03
	Belgium(3,297)	offline (16)	432 (82)	98 (18)	1.00		1.00	
		face-to-face (14)	346 (75)	112 (25)	**0.70**	**0.52,0.95**	0.78	0.57,1.07
		online (70)	1,824 (79)	485 (21)	0.85	0.67,1.08	**0.75**	**0.58,0.96**
	France(2,639)	offline (24)	358 (56)	282 (44)	1.00		1.00	
		face-to-face (8)	127 (59)	87 (40)	1.15	0.84,1.57	1.34	0.96,1.88
		online (68)	1188 (67)	597 (33)	**1.57**	**1.30,1.88**	**1.47**	**1.20,1.79**
	Italy(625)	offline (15)	44 (48)	48 (52)	1.00		1.00	
		face-to-face (15)	46 (48)	49 (52)	1.02	0.58,1.82	1.19	0.65,2.17
		online (70)	157 (36)	281 (64)	**0.61**	**0.39,0.96**	**0.59**	**0.37,0.95**
	Portugal(758)	offline (8)	40 (73)	15 (27)	1.00		1.00	
		face-to-face (33)	172 (68)	81 (32)	0.79	0.41,1.52	0.79	0.40,1.58
		online (59)	319 (71)	131 (29)	0.91	0.49,1.71	0.76	0.39,1.49
Ninfea	Italy(2,847)	offline (51)	1,385 (92)	126 (8)	1.00		1.00	
		face-to-face (33)	902 (91)	89 (9)	0.92	0.67,1.24	1.02	0.73,1.43
		online (16)	415 (87)	60 (13)	**0.60**	**0.42,0.84**	**0.68**	**0.48,0.98**
ELF	NewZealand(779)	offline (15)	80 (70)	35 (30)	1.00		1.00	
		face-to-face (83)	391 (60)	257 (40)	0.66	0.43,1.02	0.65	0.42,1.01
		online (2)	11 (69)	5 (31)	0.96	0.31,2.98	0.94	0.30,2.9

*OR, odds ratio adjusted for: age, smoking, educational level, presence of chronic disorder/condition for all the three cohorts; plus gender, household composition and vaccination against seasonal influenza for the Influenzanet cohort; plus for trimester of pregnancy for the Ninfea cohort; CI, confidence interval. Bold indicates that 1 lies outside the 95% confidence interval. Sample size refers to participants with complete data.

Despite large confidence intervals for some cohort-specific estimates, the effect of face-to-face strategies on follow-up participation was not associated with participation at follow-up when compared with offline strategies, except in the Swedish Influenzanet, where face-to-face strategies were associated with an increased participation at follow-up. Online communication compared to other offline methods was consistently associated with a lower participation in all studies but Influenzanet-France.

**Figure 1:**
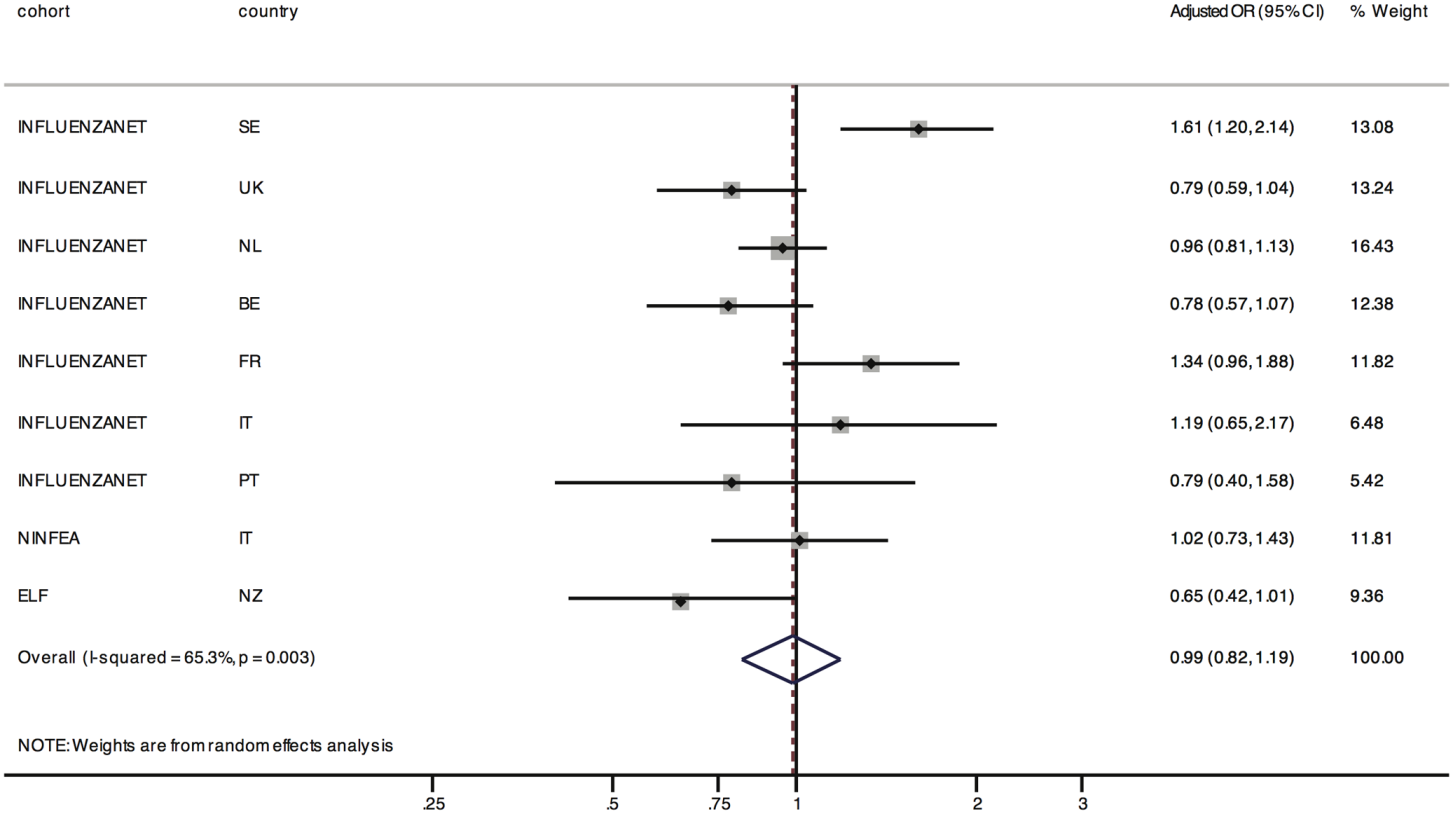
Summary of the results reported in **Table 2**. The effect of face-to-face recruitment on follow-up participation compared to offline recruitment is shown. Given that the studies are highly heterogeneous by design and target different populations, the pooled estimate should be considered as an average of the study-specific effects rather than as a causal estimate.

**Figure 2:**
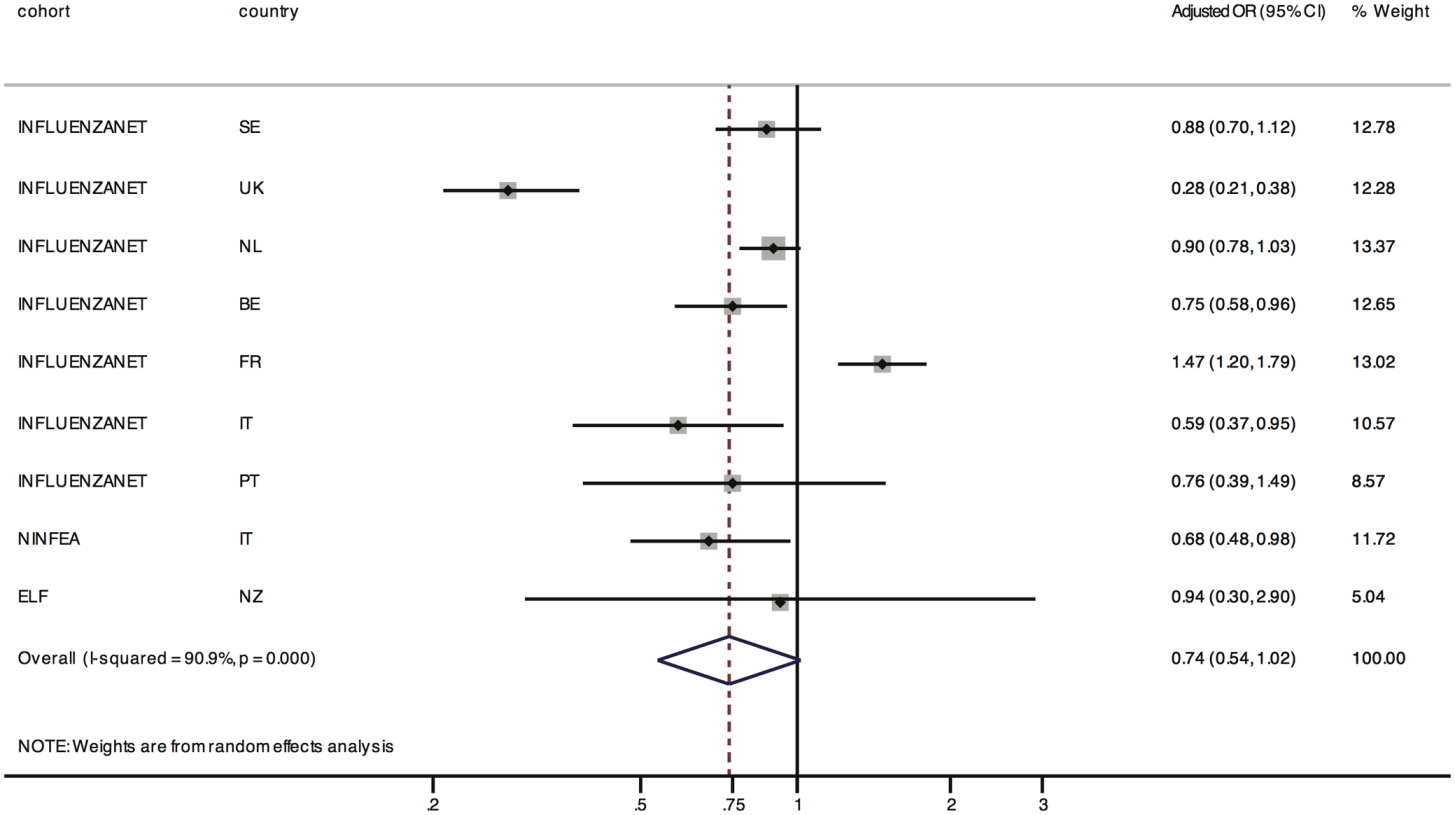
Summary of the results reported in **Table 2**. The effect of online recruitment on follow-up participation compared to offline recruitment is shown. Given that the studies are highly heterogeneous by design and target different populations, the pooled estimate should be considered as an average of the study-specific effects rather than as a causal estimate.

Figs. 1 and 2 summarize the results using a forest plot. The adjusted OR of participation at follow-up for face-to-face recruitment *vs.* offline methods was 0.99, 95% CI 0.82–1.19, with an i^2^ of 65% (analyses restricted to the the seven Influenzanet studies revealed an OR of 1.03, 95% CI 0.83,1.29, i^2^ = 69%) The pooled OR for online recruitment *vs.* offline methods was 0.74 (95% CI: 0.54,1.02), with an i^2^ of 91% (analyses restricted to the Influenzanet cohorts gave an OR of 0.74, 95% CI: 0.51,1.07, i^2^ = 93%). The high i^2^ values indicate that most of the variability across studies is due to heterogeneity. This is not unexpected due to the large differences among the studies and populations and indeed the meta-analytic estimate was presented mainly for descriptive purposes.

A percentage of individuals with missing data larger than 15% has been observed only in the French, Italian and Portuguese Influenzanet studies. Pooled estimates including only studies with a percentage of missing data lower than 15% (face-to-face vs. offline recruitment: OR = 0.94, 95% CI: 0.75, 1.18; online vs. offline recruitment: OR = 0.67, 95% CI: 0.47, 0.96) were similar to those estimated including all the studies. Analogously, unadjusted ORs estimated using the entire cohorts (data not shown) were similar to those obtained using only individuals with complete data (Table 2) suggesting that the use of a complete-case analysis to treat missing data did not bias our estimates more than marginally.

As a sensitivity analysis, we restricted the Influenzanet cohort to women aged 18–44 (fertile age). The sample size decreased to 26% of the initial cohort, but results were not modified qualitatively.

## Discussion

This is, to our knowledge, the first study investigating the association between different recruitment communication strategies and follow-up participation conducted in a variety of different Internet-based epidemiological studies. The nine studies (seven Influenzanet cohorts, the Ninfea cohort and the ELF cohort) included in this work are heterogeneous with regards to several characteristics. Influenzanet targets the general population, including a wide range of ages, while the others target only pregnant women. The outcome of interest is a seasonal infectious disease in the first case and chronic diseases in the latter studies. The period of follow-up is in the order of weeks for Influenzanet, and months for the Ninfea and ELF study. Moreover, the follow-up participation proportion is quite heterogeneous among the different studies, ranging from 43% in the Swedish Influenzanet up to 89% in the Ninfea cohort (65% on average). Communication strategies used to recruit participants in the three cohorts are quite different (TV, radio, newspapers *vs*. leaflet, posters, discussions with a medical doctor), but they can be grouped into three main categories: offline, online and face-to-face methods.

Despite these pronounced heterogeneities, and the fact that the studies targeted different populations, an overall pattern emerged: individuals recruited by means of online communication were less prone to participate to follow-up compared with individuals enrolled with offline methods. The consistency of the findings over heterogeneous studies suggests that the effects on follow-up participation of the communication strategies are likely to be generalizable to other Internet-based epidemiological studies carried out in different settings. However, as suggested by the results of the French Influenzanet cohort, there might be exceptions and, in some situations, an online communication strategy may be more effective than off-line campaigns. The differences in the efficacy of online communication strategies in France could be partially explained by a rather low participation rate at follow-up (see Table 2) of people recruited with offline methods that are used as the baseline in the multivariable regression model. However, such a low participation rate for people enrolled through offline methods has been observed also in Italy and Sweden where the OR associated with the online advertisement was below 1, meaning the online methods had a lower participation.

It is worth emphasizing that in the present analysis, some cohorts (Dutch, Belgian, French and Italian Influenzanet) recruited almost 70% of participants through online communication, highlighting the importance of such approaches in assembling the cohort. The UK Influenzanet and Ninfea cohorts received a smaller but still conspicuous contribution from the online communication campaign, which was almost negligible only for the ELF cohort. From this perspective, the unprecedented opportunities of disseminating the study on a large scale should be well balanced with the potential lower participation to follow-up of online-recruited individuals. Our findings highlight the need to conduct additional methodological research aimed at enhancing the participation to follow-up of individuals recruited by means of online media. Given the wide spectrum of online communication media, it might be important to verify whether additional prompts, either through social networks (such as Facebook or Twitter), online voice-call services (such as Skype or Google Voice), online calendar reminders, or smartphone applications would lead to higher participation at follow-up.

Behavioral science and psychological research have already pointed out the impact of culture and communication method on the spread of information and examined the interactive effects of type of media and communicator on persuasiveness of message in some special settings [19]–[22]. Given the nature of our study we are not in a position to extrapolate any conclusions on the psychological motivation behind the relationship between participants’ behavior and communication strategy, but one can speculate that the effectiveness of face-to-face communication depends on the perceived reliability of the speaker and all the issues concerning the human interactions regarding the type of relationships that occur between the two speakers. Moreover, concerning offline vs. online communication, we might conjecture that all the additional effort to remember or write down the website address and visit it after becoming aware of it (offline case) rather than visiting it in real time by following a link on a previously visited webpage (online case) can select more motivated people who will also participate at follow-up.

An additional issue that calls for deeper investigation is related to the participation in long-term follow-up. In our study we included studies with a short follow-up duration (Influenzanet cohorts, 30–60 days) and studies with a longer follow-up (Ninfea and ELF cohorts, 6–15 months). Results of the effects of the recruitment strategy were similar. However, it could be that for much longer follow-up durations, e.g. several years, a confident attitude towards Internet usage might reduce the loss to follow-up among participants who became aware of the study through an online communication strategy.

In conclusion, while more research has to be done to understand the advantages and limitations of Internet-based epidemiological studies, our results suggest that the recruitment methods may affect participant attrition in Internet-based cohort studies.

## Supporting Information

S1 Table **Data summary comparing the participants with complete versus missing data.**
(DOCX)
