# Supplementary material for: Association between Recruitment Methods and Attrition in Internet-Based Studies
Source: PLoS One. 2014 Dec 9;9(12):e114925. doi: 10.1371/journal.pone.0114925 (PMC4260912; doi:10.1371/journal.pone.0114925)
Supplement: S1 Table — Data summary comparing the participants with complete versus missing data. (DOCX) [file pone.0114925.s001.docx]

Table S1: Data summary comparing the participants with complete *versus* missing data.

| Cohort | Country (percentage of missing data) | Participation at follow up for subjects with complete data | Participation at follow up for subjects with missing data |
| --- | --- | --- | --- |
| Influenzanet | Sweden (14%) | 43% | 43% |
|  | UK (12%) | 55% | 49% |
|  | Netherlands (13%) | 77% | 71% |
|  | Belgium (14%) | 79% | 79% |
|  | France (25%) | 63% | 61% |
|  | Italy (54%) | 40% | 36% |
|  | Portugal (34%) | 70% | 64% |
| Ninfea | Italy (6%) | 92% | 68% |
| ELF | New Zealand (1%) | 62% | 33% |
